# Isotemporal substitution of sleep or sedentary behavior with physical activity in the context of frailty among older adults: a cross-sectional study

**DOI:** 10.1590/1516-3180.2021.0420.R3.03032022

**Published:** 2022-08-01

**Authors:** Giovana Silva Martins, Lucas Lima Galvão, Sheilla Tribess, Joilson Meneguci, Jair Sindra Virtuoso

**Affiliations:** I MSc. Physical Education Professional, Postgraduate Student in Physical Education, Department of Sports Sciences, Universidade Federal do Triângulo Mineiro (UFTM), Uberaba (MG), Brazil.; II MSc. Physical Education Professional, Postgraduate Student in Physical Education, Department of Sports Sciences, Universidade Federal do Triângulo Mineiro (UFTM), Uberaba (MG), Brazil.; III PhD. Physical Education Professional, Associate Professor and Coordinator, Postgraduate Course in Physical Education, Department of Sports Sciences, Universidade Federal do Triângulo Mineiro (UFTM), Uberaba (MG), Brazil.; IV PhD. Physical Education Professional Support Service and Dean, Research and Scientific Production, Universidade Federal do Triângulo Mineiro (UFTM), Uberaba (MG), Brazil.; V PhD. Physical Education Professional and Associate Professor II, Department of Sports Sciences, Universidade Federal do Triângulo Mineiro (UFTM), Uberaba (MG), Brazil.

**Keywords:** Frail elderly, Aging, Health behaviors, Public health, Sedentary behavior, Sitting time, Epidemiology, Cohort

## Abstract

**BACKGROUND:**

Frailty syndrome is associated with various physical, cognitive, social, economic, and environmental factors. Although frailty syndrome occurs progressively with age, prevention and treatment are possible. Reducing or eliminating risks and increasing protective factors may be potential strategies for reducing the prevalence of injuries related to frailty. One of the most effective actions is to decrease the time spent in sedentary behavior (SB) by increasing regular physical activity (PA).

**OBJECTIVE:**

To examine the hypothetical effect of substitution of the time spent in sleep or SB with an equivalent time spent performing moderate or vigorous PA on frailty syndrome in the older population.

**DESIGN AND SETTING:**

An analytical cross-sectional study conducted using exploratory methods of survey, carried out in Alcobaça city, Bahia, Brazil.

**METHODS:**

A total of 456 older adults of both sexes, aged ≥ 60 years, participated in this study. Frailty syndrome was identified according to the criteria of the Study of Osteoporotic Fractures. PA and SB were assessed using the International Physical Activity Questionnaire, and sleep was assessed using the Pittsburgh Sleep Quality Index. The effects of time substitution on these behaviors were verified using Poisson regression.

**RESULTS:**

The replacement of 60 min/day of SB (prevalence ratio, PR = 0.52; 95% confidence interval, CI: 0.28–0.96) or sleep (PR = 0.52; 95% CI: 0.27–0.98) with 60 min/day of moderate PA (MPA) was associated with a 48% reduction in the prevalence of frailty syndrome.

**CONCLUSIONS:**

Replacing the time spent sitting or sleeping with the same amount of MPA time may reduce frailty; the longer the duration of time spent in the substitution of sleep or SB with MPA, the greater the benefits.

## INTRODUCTION

Expansion of the older adult population is a global phenomenon. Data have shown that the number of older adults (aged ≥ 60 years) worldwide is expected to double from 841 million people in 2013 to over 2 billion in 2050.^1^ In Brazil alone, it is estimated that by 2050, older adults will comprise almost a third of the total population (29.3%).^2^

Population aging is a multifaceted challenge because health problems such as frailty syndrome are common at this stage.^3^ This impacts the individual and the society, as the associated problems directly affect the quality of life and functionality of the older person.^4^

Frailty syndrome is related to physiological alterations of the musculoskeletal, neuroendocrine, immunological, and cardiorespiratory systems^5^ mainly causing muscle loss, appetite alteration, and a chronic inflammatory state,^3^ as well as common chronic diseases such as cardiovascular diseases.^5^ In addition, frailty syndrome is associated with various physical, cognitive, social, economic, and environmental factors.^6^ It may be aggravated by the presence of one or more preexisting diseases,^7^ thereby increasing the vulnerability to adverse outcomes such as impaired physical and functional capacity, high occurrence of falls, increased use of medication, hospitalization, institutionalization, and death.^8^

Although frailty syndrome occurs progressively with age, prevention and treatment are possible. Reducing or eliminating risks and increasing protective factors are probable strategies for reducing the prevalence of frailty and frailty-related injuries.^9^ One of the most effective actions in this regard is to decrease the time spent in sedentary behavior (SB)^9^ and increase regular physical activity (PA). This helps in the reduction of symptoms of the syndrome^10^ and affects its associated parameters such as improvement in physical performance,^10^ muscular strength, mobility, body composition, and functionality and fall reduction.^11^

In the context of PA epidemiology, the isotemporal substitution model, developed by Mekary et al.,^12^ is a simple and suitable method for the analysis of PA recommendations. This analysis estimates the relative effects of time spent on different behaviors. It is a well-established and validated model and holds great relevance for public health guidelines.^12^ Previous researchers^13,14^ have used the isotemporal model approach to estimate the effects of substituting the time spent in SB with an equal amount of time spent in PA on frailty in older adults, but none of the earlier studies have included the time spent in sleep in this model. This variable deserves attention, as there is evidence that both long and short sleep durations are associated with frailty.^15^ Furthermore, the time spent in the substitution of SB with PA and the intensity of PA were important indicators of frailty in the current study. Prior information about these factors is crucial while imparting recommendations on PA to older adults, considering that they represent a population segment that shows low participation in PA. In particular, older adults from socioeconomically disadvantaged backgrounds maintain inadequate levels of PA.^16^

## OBJECTIVE

The aim of this study was to examine the hypothetical effects of substituting the time spent in sleep or SB with the same amount of time spent in performing moderate or vigorous PA on frailty syndrome in an older adult population.

## METHODS

### Participants and study design

This observational, analytical, and cross-sectional study was conducted using exploratory survey methods on older adults of both sexes (aged ≥ 60 years) in the state of Bahia, Brazil, as part of the project Longitudinal Study of the Elderly Health of Alcobaça (Estudo Longitudinal de Saúde do Idoso de Alcobaça [ELSIA]). The study details, data collection procedures, and inclusion criteria have been described previously.^17^

Initially, the present study consisted of 743 older adults registered in the Family Health Strategy in Alcobaça City. The Family Health Strategy aims to reorganize primary health care in the country. It has been devised for the purpose of expansion and consolidation of primary care. Its objective is to reorient the work process using a cost-effective and multidisciplinary approach in order to develop the principles, guidelines, and fundamentals of primary care, so that it results in a resolute impact on the health situation of people and communities. During data collection, 54 older individuals refused to participate in the study, 58 were excluded for not meeting the inclusion criteria, and 158 older individuals were excluded after three failed attempts to contact them. Of the 473 participants aged ≥ 60 years included in the study, 17 did not fulfill at least one of the frailty criteria. Thus, the final sample consisted of 456 older adults (172 [37.7%] males and 284 [62.3%] females) with a mean age of 70.25 years (± 8.25 years).

The exclusion criteria were as follows: severe cognitive impairment (≤11 points) according to the Mini Mental State Examination (MMSE) guidelines adapted for the Brazilian population,^18^ severely impaired visual and hearing acuity, use of wheelchairs, severe sequelae of stroke with localized loss of strength, or having a terminal illness.

For home visits, the researchers used data provided by the Municipal Health Department of Alcobaça as a reference. Contact was made with older adults through home visits, informing them of the objectives, and requesting their participation in the research on a voluntary basis.

The study protocol and procedures were in accordance with the Declaration of Helsinki and approved by the Human Research Ethics Committee of the Universidade Federal do Triângulo Mineiro (UFTM) on February 27, 2015 (ethics code: 966.983).

### Frailty assessment

Frailty syndrome was identified on the basis of three criteria proposed by the Study of Osteoporotic Fractures (SOF): 1) self-reported unintentional weight loss of 4.5 kg or more in the past year; 2) self-reported fatigue, as assessed by questions on the Geriatric Depression Scale (GDS-15), e.g., “Did you stop performing many of your activities and interests?” and “Do you feel you are full of energy?”. A positive answer to the first question and/or a negative answer to the second question were considered signs of lack of energy/low resistance; and 3) loss of strength, defined as the inability to sit down and stand up five times from a chair, without using the arms, according to the test guidelines. Older adults who satisfied two or three of these criteria were classified as frail, whereas the others were classified as non-frail.^19^

### Behavioral variables

PA and SB were assessed using the International Physical Activity Questionnaire (IPAQ), which has been customized for the Brazilian older adult population.^20,21^ The intensity of PA was determined based on moderate physical activity (MPA) and vigorous physical activity (VPA) performed for at least 10 continuous minutes during a typical weekday and in different domains such as work, transport, recreation/leisure, and housework. The population was dichotomized as sufficiently active (≥ 150 min/week of MPA, 75 min/week of VPA, or a combination of both) and insufficiently active.^22^

SB was assessed by asking questions regarding the time spent sitting on a usual day of the week (“How much time in total do you spend sitting during a weekday?”) or on a typical day of the weekend (“How much time in total do you spend sitting during one day of the weekend?”). An SB score was considered high if it was greater than the cut-off (calculated to be equivalent to 527.50 minutes/day based on the 75^th^ percentile of the SB score set). Previous research also suggests that individuals in the highest quartile of sitting time are at maximum risk of adverse health outcomes.^17,23^

The measurement of nocturnal sleep time was performed using the Pittsburgh Sleep Quality Index, modified for Brazilians (PSQI-BR),^24^ by asking the question, “During the past month, how many hours did you sleep every night?”. This measure was used to calculate the total time spent doing daily activities.

For the isotemporal adjustment models, the total durations of continuous MPA, VPA, SB, and sleep, expressed in minutes per day (minutes/day), were used.

### Covariates

Data on sociodemographic variables such as sex (male and female), age group (60-79 years and 80 years or older), marital status (not married, married, widowed, and divorced), level of literacy (not literate and literate), and number of falls in the last 12 months (0 to 3 falls and 4 or more) were collected to characterize the sample.

### Statistical analyses

The database was created using Epidata software, version 3.1b (EpiData, Odense, Denmark), and the analyses were performed using the statistical software SPSS 23.0 (IBM, Armonk, New York, United States).

Using descriptive statistics, absolute and relative frequencies and dispersion values for PA, SB, and frailty were calculated. For analyzing the association between frailty syndrome and its covariates, inferential statistics (chi-squared test) were used.

To verify the hypothetical effect of the reallocation of time spent in sleep and SB to performing PA on frailty syndrome, the isotemporal substitution approach was used.^12^ For the isotemporal replacement analyses, Poisson regression with robust variance was used for estimating the adjusted prevalence ratios (PR) and calculating the respective 95% confidence intervals (CI) for statistical significance. The effects of reallocating 5, 10, 15, 20, 25, 30, 35, 40, 45, 50, 55, and 60 min spent on sleep and SB to MPA and VPA on the presence of frailty syndrome were verified. The models were adjusted for sex, age, number of falls, literacy level, and marital status. A significance level of P < 0.05 was adopted.

## RESULTS

The study sample consisted of 456 adults of both sexes aged ≥ 60 years. The prevalence of frailty syndrome among the older adults was found to be 8.6% (n = 39). Frailty was associated with time of exposure to SB (P = 0.006) and PA (P = 0.014), as shown in Table 1.

**Table 1.** Sociodemographic, health-related, and behavioral characteristics associated with frailty syndrome

Variables	Total n (%)	Frailty syndrome		
		Not frail	Frail	P
		n (%)	n (%)	
**Sex**				
Male	172 (37.7)	158 (91.9)	14 (8.1)	0.476
Female	284 (62.3)	259 (91.2)	25 (8.8)	
**Age range**				
60 to 79 years old	388 (85.1)	358 (92.3)	30 (7.7)	0.107
80 years or older	68 (14.9)	59 (86.8)	9 (13.2)	
**Marital status**				
Not married	41 (9.0)	39 (95.1)	2 (4.9)	0.117
Married	214 (46.9)	201 (93.9)	14 (6.1)	
Widowed	121 (26.5)	108 (89.3)	13 (10.7)	
Divorced	80 (17.5)	69 (86.3)	11 (13.8)	
**Number of falls**				
0 to 3 falls	425 (93.2)	391 (92.0)	34 (8.0)	0.114
4 or more	31 (6.8)	26 (83.9)	5 (16.1)	
**Literacy level**				
Not literate	146 (32.2)	134 (91.8)	12 (8.2)	0.501
Literate	308 (67.8)	281 (91.2)	27 (8.8)	
**Physical activity level**				
Physically active	246 (53.9)	232 (94.3)	14 (5.7)	0.014
Insufficiently active	210 (46.1)	185 (88.1)	25 (11.9)	
**Sedentary behavior P**_**75**_				
< 527.50 min/day	343 (75.2)	321 (77.0)	22 (6.4)	0.006
≥ 527.50 min/day	113 (24.8)	96 (85.0)	17 (15.0)	

min = minutes.

The average time spent on the measured behavioral variables is shown in Table 2.

**Table 2.** Time spent in different behaviors adopted by the older adults throughout the day

	Average (SD)	Median (IQR)
Moderate physical activity (min/day)	45.05 (61.76)	22.86 (54.28)
Vigorous physical activity (min/day)	4.16 (24.41)	00.00 (00.00)
Sleep (min/day)	434.20 (104.07)	420.00 (138.75)
Sedentary behavior (min/day)	426.91 (157.02)	418.57 (205.18)

SD = standard deviation; IQR = interquartile range; min = minutes.

In the isotemporal substitution analysis (Table 3), for all the durations of time spent in SB or sleep, MPA, and VPA, it was observed that the replacement of MPA with sleep or SB resulted in a higher probability of frailty syndrome (P < 0.05). However, the replacement of SB or sleep time with MPA had a protective effect, and longer durations of replacement corresponded with greater protective effects. Replacing 60 min/day of SB or sleep with 60 min/day of MPA was associated with a 48% reduction in the likelihood of frailty.

**Table 3.** Isotemporal substitution model of time spent in sleep, sedentary behavior, and physical activity along with the prevalence ratios of frailty syndrome

Replacement templates	Frailty syndrome			
	PR (95% CI)	PR (95% CI)	PR (95%CI)	PR (95% CI)
	MPA	VPA	Sleep	SB
**5 minutes**				
MPA replacement	–	1.03 (0.94-1.12)	1.05 (1.00-1.11)*	1.05 (1.00-1.11)*
VPA replacement	0.96 (0.88-1.05)	–	1.02 (0.95-1.08)	1.02 (0.95-1.08)
Sleep replacement	0.94 (0.89-0.99)*	0.98 (0.92-1.04)	–	1.00 (0.98-1.01)
SB replacement	0.94 (0.90-0.99)*	0.98 (0.92-1.04)	1.00 (0.98-1.01)	–
**10 minutes**				
MPA replacement	–	1.07 (0.89-1.27)	1.11 (1.00-1.23)*	1.11 (1.00-1.23)*
VPA replacement	0.93 (0.78-1.11)	–	1.04 (0.91-1.18)	1.04 (0.91-1.17)
Sleep replacement	0.89 (0.80-0.99)*	0.96 (0.84-1.09)	–	1.00 (0.97-1.02)
SB replacement	0.89 (0.81-0.99)*	0.96 (0.84-1.08)	1.00 (0.97-1.02)	–
**15 minutes**				
MPA replacement	–	1.10 (0.85-1.44)	1.17 (1.00-1.37)*	1.17 (1.00-1.37)*
VPA replacement	0.90 (0.65-1.17)	–	1.06 (0.87-1.27)	1.06 (0.88-1.27)
Sleep replacement	0.85 (0.72-0.99)*	0.94 (0.78-1.13)	–	1.00 (0.95-1.04)
SB replacement	0.85 (0.73-0.99)*	0.94 (0.78-1.13)	1.00 (0.96-1.04)	–
**20 minutes**				
MPA replacement	–	1.14 (0.80-1.62)	1.24 (1.00-1.52)*	1.24 (1.01-1.52)*
VPA replacement	0.87 (0.61-1.23)	–	1.08 (0.84-1.39)	1.08 (0.84-1.38)
Sleep replacement	0.80 (0.65-0.99)*	0.92 (0.71-1.18)	–	1.00 (0.94-1.05)
SB replacement	0.80 (0.65-0.98)*	0.92 (0.72-1.18)	1.00 (0.94-1.05)	–
**25 minutes**				
MPA replacement	–	1.18 (0.76-1.83)	1.31 (1.00-1.69)*	1.31 (1.01-1.69)*
VPA replacement	0.84 (0.54-1.30)	–	1.10 (0.80-1.51)	1.10 (0.80-1.51)
Sleep replacement	0.76 (0.58-0.99)*	0.90 (0.66-1.24)	–	1.00 (0.93-1.07)
SB replacement	0.76 (0.59-0.98)*	0.90 (0.66-1.23)	1.00 (0.93-1.07)	–
**30 minutes**				
MPA replacement	–	1.22 (0.72-2.07)	1.38 (1.00-1.89)*	1.38 (1.01-1.87)*
VPA replacement	0.81 (0.48-1.37)	–	1.12 (0.77-1.63)	1.12 (0.77-1.63)
Sleep replacement	0.72 (0.52-0.99)*	0.88 (0.60-1.29)	–	1.00 (0.92-1.08)
SB replacement	0.72 (0.53-0.98)*	0.88 (0.61-1.29)	1.00 (0.92-1.08)	–
**35 minutes**				
MPA replacement	–	1.27 (0.69-2.34)	1.46 (1.01-2.11)*	1.46 (1.02-2.08)*
VPA replacement	0.78 (0.42-1.46)	–	1.15 (0.73)	1.15 (0.74-1.78)
Sleep replacement	0.68 (0.47-0.99)*	0.87 (0.56-1.35)	–	1.00 (0.90-1.10)
SB replacement	0.68 (0.48-0.97)*	0.87 (0.56-1.34)	1.00 (0.90-1.10)	–
**40 minutes**				
MPA replacement	–	1.31 (0.65-2.64)	1.54 (1.01-2.35)*	1.54 (1.02-2.31)*
VPA replacement	0.76 (0.37-1.53)	–	1.17 (0.70-1.94)	1.17 (0.70-1.93)
Sleep replacement	0.64 (0.42-0.99)*	0.85 (0.51-1.41)	–	1.00 (0.89-1.11)
SB replacement	0.64 (0.43-0.97)*	0.85 (0.52-1.40)	1.00 (0.89-1.11)	–
**45 minutes**				
MPA replacement	–	1.35 (0.61-2.98)	1.62 (1.01-2.60)*	1.62 (1.02-2.56)*
VPA replacement	0.73 (0.33-1.59)	–	1.19 (0.68-2.08)	1.19 (0.68-2.08)
Sleep replacement	0.61 (0.38-0.99)*	0.83 (0.47-1.47)	–	1.00 (0.88-1.13)
SB replacement	0.61 (0.38-0.97)*	0.83 (0.47-1.46)	1.00 (0.88-1.13)	–
**50 minutes**				
MPA replacement	–	1.20 (0.58-3.36)	1.71 (1.01-2.90)*	1.71 (1.03-2.85)*
VPA replacement	0.70 (0.29-1.69)	–	1.21 (0.64-2.28)	1.21 (0.65-2.26)
Sleep replacement	0.58 (0.34-0.98)*	0.82 (0.43-1.54)	–	1.00 (0.87-1.14)
SB replacement	0.58 (0.35-0.97)*	0.82 (0.44-1.53)	1.00 (0.87-1.14)	–
**55 minutes**				
MPA replacement	–	1.46 (0.56-3.83)	1.81 (1.01-3.25)*	1.81 (1.03-3.19)*
VPA replacement	0.68 (0.26-1.78)	–	1.24 (0.62-2.50)	1.24 (0.62-2.48)
Sleep replacement	0.55 (0.30-0.98)*	0.80 (0.40-1.61)	–	1.00 (0.85-1.16)
SB replacement	0.55 (0.31-0.96)*	0.80 (0.40-1.59)	1.00 (0.85-1.16)	–
**60 minutes**				
MPA replacement	–	1.51 (0.53-4.31)	1.91 (1.01-3.61)*	1.91 (1.03-3.53)*
VPA replacement	0.66 (0.23-1.91)	–	1.27 (0.59-2.73)	1.27 (0.59-2.71)
Sleep replacement	0.52 (0.27-0.98)*	0.79 (0.37-1.68)	–	1.00 (0.84-1.18)
SB replacement	0.52 (0.28-0.96)*	0.79 (0.37-1.66)	1.00 (0.84-1.18)	–

CI = confidence interval; PR = prevalence ratio; MPA = moderate physical activity; VPA = vigorous physical activity; SB = sedentary behavior. PR adjusted for sex, age group, number of falls, literacy level, and marital status. *P < 0.05.

The replacement of VPA with SB or sleep time, on the other hand, did not show association with frailty syndrome in the older adults, for all durations of time tested.

## DISCUSSION

This study showed the hypothetical effect of reallocation of time between activities that require different intensities of movement (light or no movement to vigorous movement) on the prevalence of frailty among older adults. The results showed that replacing time spent on SB or sleep with MPA results in easing of symptoms of the frailty syndrome.

Worldwide, older adults are generally recommended to modify their lifestyle to include low levels of PA. In spite of this, it has been noticed that there are limited access to information and less opportunities in regions of low socioeconomic status, and the older adults from such backgrounds usually maintain low levels of PA. On the other hand, information is easily accessible in large urban centers, and the older adults are physically active as well.^25^ The population studied in this investigation resides in the municipality of Alcobaça (Brazil), with an average Human Development Index of 0.608.^26^ It is a regional population, and its characteristics differentiate it from the populations residing in more developed and/or populous cities, which were included in other national studies.^27,28^

The prevalence of frailty found in this research (8.6%) is similar to the worldwide prevalence of frailty in the population aged 65 and above, which is equal to 10.7% (95% CI = 10.5–10.9).^29^ It is important to note that the prevalence of frailty varies widely (from 4.0 to 59.1%), depending on the population and the operationalization of frailty according to the method of analysis.^29^ In the Lafaiete Coutinho region, also located in the state of Bahia (Brazil), a prevalence of frailty of 23.8% was identified.^30^ This discrepancy between prevalence of frailty in Alcobaça and Lafaiete Coutinho can be explained by the fact that the former is a coastal region, where people are comparatively more active than people living in non-coastal cities.^31,32^ Another reason might be that frailty was assessed using different criteria (SOF for the Alcobaça population and Fried Phenotype for the Lafaiete Coutinho population).

Sleep time was a differential variable in this study, and it has not been included in analysis using the isotemporal replacement model by any previous study.^13,14,33^ In analyses of sleep-related mortality, a long duration of sleep (≥ 9 h) was shown to be a confounding factor and may be indicative of frailty among older adults. Excessive sleep time reduces the time available for active behavior, and prolonged sleep time can lead to several health risks.^34^ On the other hand, short sleep time (≤ 6 hours) is also associated with frailty among older adults, as it is related to a decrease in gait speed and symptoms of exhaustion,^15^ both of which are components of frailty.

The relationship between sleep disorders and frailty syndrome can be explained by several physiological mechanisms. Inadequate sleep results in oxidative stress, imbalance between the levels of anabolic and catabolic hormones, and acceleration of processes such as sarcopenia.^35^ Short sleep durations and related disturbances are associated with the proliferation of inflammatory cells and an increase in the concentration of adipokines,^35^ both of which are determinant factors in the physiopathologic development of frailty syndrome.^36^ Besides this, sleep deprivation also modifies the levels of stress response markers such as cortisol and norepinephrine.^37^ On the other hand, excess of sleep also impairs cognitive functions and leads to reduction of PA.^15^ Consequently, the decrease in energy expenditure contributes to the elevation of the degree of adiposity and insulin resistance and increase in the concentrations of interleukin 6 (IL-6) and C-reactive protein (CRP) in blood plasma.^38^

In practice, it is necessary to be cautious while reallocating time between different variables. Older adults who usually sleep for very short durations would not be enthusiastic about substituting sleep for PA, because this would further limit the sleep time available to them, which in turn might cause many health-related complications.^15,35,37^ Therefore, the recommendation of substitution of sleep for PA should be directed only towards older adults with a high average sleep time.

The combination of insufficient level of PA and longer duration of time spent in SB leads to caloric overload and accumulation of central adipocytes, which downregulate the production of anti-inflammatory adipokines.^39^ In addition, SB negatively affects lipid and glucose metabolism and deregulates hemodynamic balance of the lower extremities.^40^ Impairment of all these functions leads to an inflammatory state, which contributes to the development of frailty syndrome.^41^ However, regular PA reduces systemic inflammation.^42^ This highlights the importance of discontinuing SB and incorporating regular PA in the lifestyle in order to reduce the symptoms of frailty.

Song et al.^43^ investigated a US cohort in which the participants were free of physical frailty at the baseline level. The participants spent an average of 9.9 hours/day in SB and less than 20 min in MPA, and it was found that those who spent more time in SB had a higher risk of developing frailty syndrome. A higher percentage of SB was strongly associated with a higher risk of frailty (risk relative, RR = 1.55 for a 10% increase; 95% CI = 1.04-2.32), regardless of participation in MPA and other controlled risk factors.^43^ However, Mañas et al.^44^ reported that moderate and vigorous PA (MVPA) had a moderating effect on the relationship between SB and frailty. The authors showed that spending 27 min/day on MVPA eliminated the increased risk of SB-associated frailty among older adults, affirming the importance of engaging in activities like MVPA, especially for insufficiently active individuals.

A previous research revealed that isotemporal replacement of 30 min of SB with light physical activity (LPA) decreased the risk of frailty among older adults.^13^ These findings indicated that increasing PA of lower intensity is feasible for the target population. Along similar lines, another study showed that replacement of at least 113 minutes of SB with LPA or 41 minutes of SB with MVPA per day resulted in a decrease in the frailty index.^33^ However, it is known that reaching the daily PA recommendation is still a challenge for most older adults.^45^ Health benefits are primarily obtained on increasing the level of PA above the baseline.^46^ Overall, these reports corroborate our results in the current study, which showed that the risk of frailty is reduced only when sleep time or SB is replaced by MPA (not VPA). Even small modifications (5 min/day of MPA) in the lifestyle are beneficial for older adults.

Earlier studies have also shown that performing LPA and MPA rather than VPA contributes to better physical and mental health in older adults.^47^ Generally, older adults show very limited participation in VPA because of multiple factors such as orthopedic problems, arthritis, and cardiovascular diseases. In particular, older adults refrain from VPA when it has to be performed for a long period of time. The participants in our study also indulged in a very limited amount of VPA, apparently because data on at least 10 continuous minutes of activity were used for the evaluation of PA.

This study has some limitations and implications that should be taken into consideration. These results have been obtained by statistical analysis of the data available from a cross-sectional study considering multiple variables simultaneously. However, these results should not be used to predict the cause and effect or values of the behavior variables for the long term. Moreover, these findings may not be comparable with the results obtained from studies that have used non-identical methods of data collection and calculation because the underlying variables might be different. In addition, since the subjective measures of PA and SB levels were self-reported by the participants, they tended to be overestimated and underestimated, respectively.

The main highlights of this study are as follows: the inclusion of sleep time in the estimation of the hypothetical isotemporal substitution effect, which could be more effective in explaining the behavior substitution for the measured hours in a day than the inclusion of time spent on SB alone. Further analysis on a larger sample size is required for better understanding of the outcome of frailty, and the different durations and intensities of PA in the isotemporal substitution model offer a broader scope for the verification of their effects in the context of frailty.

## CONCLUSION

Replacing sitting or sleeping time with the same amount of MPA time can reduce the risk of frailty syndrome; the longer the reallocation time, the greater the benefits, which may equate to almost 91% risk reduction.

By understanding the necessities and specificities of health behavior in older adults, health care professionals can make appropriate decisions about the most suitable recommendations and interventions needed for the promotion and maintenance of health of the older adult population.
